# Long non-coding RNA-dependent transcriptional regulation in neuronal development and disease

**DOI:** 10.3389/fgene.2014.00164

**Published:** 2014-06-06

**Authors:** Brian S. Clark, Seth Blackshaw

**Affiliations:** ^1^Solomon Snyder Department of Neuroscience, Johns Hopkins University School of MedicineBaltimore, MD, USA; ^2^Department of Ophthalmology, Johns Hopkins University School of MedicineBaltimore, MD, USA; ^3^Department of Neurology, Johns Hopkins University School of MedicineBaltimore, MD, USA; ^4^Center for High-Throughput Biology, Johns Hopkins University School of MedicineBaltimore, MD, USA; ^5^Institute for Cell Engineering, Johns Hopkins University School of MedicineBaltimore, MD, USA

**Keywords:** cell fate, neurogenesis, embryonic stem cells, neural stem cells, transcription factors, epigenetics, long noncoding RNA, molecular scaffold

## Abstract

Comprehensive analysis of the mammalian transcriptome has revealed that long non-coding RNAs (lncRNAs) may make up a large fraction of cellular transcripts. Recent years have seen a surge of studies aimed at functionally characterizing the role of lncRNAs in development and disease. In this review, we discuss new findings implicating lncRNAs in controlling development of the central nervous system (CNS). The evolution of the higher vertebrate brain has been accompanied by an increase in the levels and complexities of lncRNAs expressed within the developing nervous system. Although a limited number of CNS-expressed lncRNAs are now known to modulate the activity of proteins important for neuronal differentiation, the function of the vast majority of neuronal-expressed lncRNAs is still unknown. Topics of intense current interest include the mechanism by which CNS-expressed lncRNAs might function in epigenetic and transcriptional regulation during neuronal development, and how gain and loss of function of individual lncRNAs contribute to neurological diseases.

## IDENTIFICATION, CONSERVATION, AND DIVERSITY OF lncRNAs

Annotation and high-throughput deep sequencing of the transcriptomes of multiple species have led to the belief that much of the genome is transcribed; however, only a minority of transcribed sequences contain evolutionarily conserved open reading frames (Okazaki et al., 2002; Maeda et al., 2006; Kapranov et al., 2007, 2010; Derrien et al., 2012; Dunham et al., 2012). Many of the transcribed sequences are thus unlikely to encode proteins. Among all human non-coding transcripts, at least 10,000 are estimated to be >200 nucleotides, and are accordingly designated as long non-coding RNAs (lncRNAs; Derrien et al., 2012). Based on transcriptome analysis of protein coding genes (Okazaki et al., 2002), transcripts are typically classified as lncRNAs when they do not contain any open reading frame >100 amino acids in length. Although few lncRNAs contain ORFs longer than predicted by pure chance (Dinger et al., 2008, 2011), they also show relatively low levels of evolutionary conservation overall, suggesting that they may encode short, evolutionarily divergent proteins similar to those observed in *Drosophila* (Kondo et al., 2010). Recently, researchers detected a number of evolutionarily conserved sequences that do encode small proteins through both ribosome profiling and mass spectrometry (Bazzini et al., 2014). However, analysis of other mass spectrometry experiments reveals that lncRNAs rarely produce detectable protein products (Banfai et al., 2012; Slavoff et al., 2013). Furthermore, ribosome profiling experiments have indicated that while lncRNAs can associate with ribosomes, ribosome occupancy of lncRNAs displays features more congruent with untranslated regions (5′ UTRs) and other classical ncRNAs, such as small nucleolar RNAs (snoRNAs) and microRNAs (miRNAs; Guttman et al., 2013). Combined with data showing that a large fraction of lncRNA transcripts are retained in the nucleus (Derrien et al., 2012), it suggests that lncRNAs impart functions as RNA transcripts.

LncRNAs are distinguished from other ncRNAs subtypes by several different features. Inherent to the name, lncRNAs are classified as such based on a length of >200 nucleotides, distinguishing them from many ncRNAs including miRNAs, snoRNAs, and others. They are also distinct from transfer RNAs (tRNAs) as they are typically transcribed by RNA polymerase II (RNA Pol II), as opposed to RNA Pol III. Moreover, lncRNAs share many features with protein-coding messenger RNAs (mRNAs) – they are capped and polyadenylated. Many lncRNAs also contain multiple exons and are subjected to alternative splicing. However, in comparison to protein-coding transcripts, lncRNAs are roughly one-third as long, contain fewer exons (~2.8 exons in lncRNAs compared to 11 exons for protein coding genes), and are expressed at 10-fold lower levels on average (Guttman et al., 2010; Cabili et al., 2011; Pauli et al., 2012). In addition, lncRNAs show a higher degree of tissue-specific expression than do protein-coding genes (Cabili et al., 2011). Compared to protein-coding genes, retrotransposon sequences and tandem repeat elements are more frequently included in lncRNA sequences (Ulitsky et al., 2011; Kelley and Rinn, 2012). These elements have been proposed to facilitate lncRNA function through either base pairing with other RNAs with similar repeat sequences, or through as yet unidentified mechanisms (Gong and Maquat, 2011; Carrieri et al., 2012).

**FIGURE 1:**
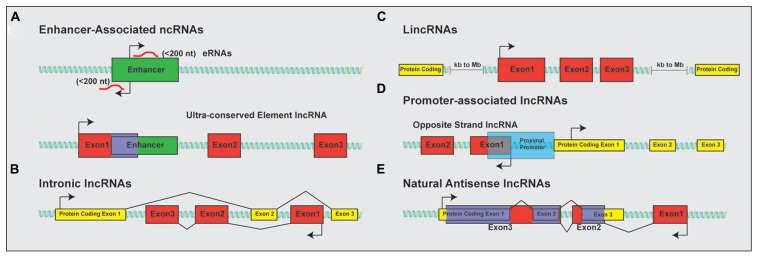
**Classification of lncRNAs based on genomic localization**. Schematic examples of the classification of lncRNAs based on genomic localization. **(A)** Enhancer-associated RNAs result from direct, bi-directional transcription of enhancer elements Ultra-conserved enhancer elements are frequently transcribed as part of lncRNA sequences. **(B)** Intronic lncRNAs localize to the introns of protein-coding genes and are transcribed from the anti-sense (pictured) or sense strand (not shown). **(C)** LincRNAs are localized to gene deserts, far removed from proximal promoter elements from neighboring protein-coding genes. **(D)** Promoter associated lncRNAs are transcribed from segments within proximal promoters of the associated protein-coding gene on the anti-sense (opposite strand lncRNAs; shown) or sense (not shown) strand relative to the protein-coding gene. **(E)** Natural antisense lncRNAs are transcribed for the antisense strand of protein-coding genes and contain complementary sequences to segments of the mature mRNA. Protein-coding exons shown in yellow; lncRNA exons shown in red; overlapping sequence shown in purple.

The discovery that much of the genome is transcribed bi-directionally has led to a diverse and still not fully standardized categorization of lncRNAs based on genomic localization. Included in the class of lncRNAs are enhancer-related lncRNAs (eRNAs) or transcribed ultra-conserved region lncRNAs (**Figure 1A**), intronic lncRNAs (**Figure 1B**), large/long intergenic or intervening non-coding RNAs (lincRNAs; **Figure 1C**), promoter associated lncRNAs (**Figure 1D**), and natural antisense transcripts (NATs; **Figure 1E**). LincRNAs have been identified through examination of sequencing reads that map expressed transcripts without clearly defined ORFs to intergenic regions. These lincRNAs usually also possess signatures of active transcription including H3K4me3, polyadenylation signals, and RNA polymerase II occupancy (Guttman et al., 2009). LncRNAs not localized to intergenic regions have been less readily identified and originally described as transcription “noise” due to overlap with protein-coding transcripts or known DNA-regulatory elements such as enhancers.

Reports suggest that intronic lncRNAs, which comprise up to 35% of non-coding transcripts, form the largest single class of lncRNAs (Birney et al., 2007; St Laurent et al., 2012). Although intronic lncRNAs were originally thought to be unprocessed pre-mRNAs of protein coding genes, current estimates suggest that up to 80% of protein coding loci have transcriptionally active introns that are expressed independently from the protein coding pre-mRNA (Dermitzakis et al., 2005; Louro et al., 2008; St Laurent et al., 2012). Further confirmation of the presence of intronic lncRNAs comes from reports that find many intronic lncRNAs localized to the cytoplasm, excluding the possibilities that intronic lncRNAs are the result of genomic DNA or unspliced pre-mRNA contamination in deep sequencing studies (Kampa et al., 2004; Kapranov et al., 2007; Mercer et al., 2008). Intronic lncRNAs are transcribed from either the sense or antisense strand of the protein-coding gene in which they are encoded, further supporting independent transcriptional regulation (Rinn et al., 2003; Bertone et al., 2004; Kampa et al., 2004).

A relatively new class of ncRNAs, eRNAs, result from bidirectional transcription of enhancers. These sequences display H3K4me1 and H3K27ac modifications, and p300/CBP and RNA polymerase II occupancy, and thus show signatures of open or poised chromatin (Heintzman et al., 2007, 2009; Visel et al., 2009; De Santa et al., 2010; Kim et al., 2010; Orom et al., 2010). While many eRNAs are short (<200 nt), there are a considerable number of lncRNAs that display lincRNA-like chromatin signatures and overlap known enhancer sequences. Because of the shared enhancer sequence, these have been further classified as transcribed ultra-conserved region-associated lncRNAs to distinguish them from shorter eRNAs.

NATs, on the other hand, were identified in bacteria and eukaryotes in the 1990s (Wagner and Simons, 1994; Vanhee-Brossollet and Vaquero, 1998). More recent studies indicate that 50–70% of protein coding genes are also transcribed in the antisense direction, with half of these antisense transcripts being non-coding (Carninci et al., 2005; Katayama et al., 2005; Galante et al., 2007). Studies have shown that many NATs display localized expression patterns that correspond inversely with their sense transcript counterparts, suggesting possible negative regulation of sense transcripts by NATs (Vanhee-Brossollet and Vaquero, 1998; Alfano et al., 2005). In contrast, many lncRNAs without overlapping sequence display expression patterns that correlate with nearby protein-coding transcripts (Luo et al., 2013).

Despite the tremendous diversity of lncRNAs, their functional importance has been underappreciated and relatively understudied, in part due to the fact that they often fail to show clear evolutionary conservation (Ulitsky et al., 2011; Basu et al., 2013). However, previous comparative genomic analyses have identified thousands of non-coding intergenic and intronic ultra-conserved sequence elements (UCEs) in the human genome (Bejerano et al., 2004; Sandelin et al., 2004). Analysis of the genomic localization of UCEs shows that UCEs are preferentially localized to loci encoding DNA-binding proteins (Sandelin et al., 2004). A recent study that incorporated transcriptome data from many different vertebrate species revealed that 4–11% of lncRNAs are conserved across the vertebrate lineage, and many of these map to UCE loci (Ulitsky et al., 2011; Basu et al., 2013). Additionally, although the primary sequence of lincRNAs that are localized in close proximity to protein-coding genes often shows little sequence conservation, synteny between vertebrate lincRNAs and protein-coding genes is often conserved during vertebrate evolution (Ulitsky et al., 2011; Qu and Adelson, 2012a, b). Combined, this suggests that the synteny and evolutionary conservation of these non-coding elements helps facilitate the regulated expression of transcription factors through enhancer activity, functional ncRNA transcripts, or both.

Analysis of sequence conservation within transcribed and regulatory regions of individual lncRNAs suggest that the proximal promoters display highest levels of evolutionary conservation (Carninci, 2007; Ponjavic et al., 2007; Marques and Ponting, 2009; Chodroff et al., 2010). Peak conservation is observed ~43 bp upstream of the transcription start site, similar to the level of conservation seen across mouse and human protein coding genes (Taylor et al., 2006; Chodroff et al., 2010). Furthermore, exonic sequence of lncRNAs is more highly conserved than intronic sequence, with exon splice sites showing highest evolutionary constraint (Chodroff et al., 2010). Short sequences within the lncRNAs are also frequently conserved. Stringent identifications of miRNAs localized within lncRNA sequence identified 97 lncRNAs that function as potential precursors to miRNA clusters (He et al., 2008). These miRNA sequences display a minimum 98% homology between rat and mouse, a far greater sequence conservation than observed for lncRNAs as a whole (He et al., 2008).

Quantification of the number of lncRNAs present across multiple species has elicited a wide range of estimates for the number of vertebrate lncRNAs. Stringent estimates suggest that 1133 lncRNAs are expressed during zebrafish development (Pauli et al., 2012). Consistent with an evolutionary increase in number, size, and divergence of regulatory elements as species become more complex (Mazumder et al., 2003; Frith et al., 2005; Taft et al., 2007), conservative estimates from recent GENCODE sequencing builds in mouse and humans (July, 2013) indicate the presence of 4074 and 13,870 lncRNAs, respectively (Derrien et al., 2012; Harrow et al., 2012). Estimates from mouse suggest that 849 of the 1328 lncRNAs examined by *in situ* hybridization show specific expression patterns in the adult brain (Mercer et al., 2008). More comprehensive analysis using RNA deep-sequencing technologies will help further elucidate and identify the exact number of lncRNAs expressed during neuronal development.

Of the 13,870 identified human lncRNAs, approximately one-third are unique to the primate lineage (Derrien et al., 2012), suggesting that ncRNA-dependent regulation of brain development may have contributed to the evolution of higher cognitive functions (Barry and Mattick, 2012; Barry, 2014). Consistent with this idea, 47 of 49 conserved sequences across evolution displayed sequence substitution rates statistically higher between human and chimpanzees than rates compared to other sequences across amniote evolution (Pollard et al., 2006). Of these human accelerated regions (HARs) that are non-coding, a quarter of these mapped to locations adjacent to genes that regulate neural development (Pollard et al., 2006). HAR1F, the most rapidly evolving sequence of all, encodes a lncRNA that is prominently expressed in the developing and adult brain. Although the function of HAR1F is still unknown, this presents a tantalizing link between lncRNAs and the formation of the proportionally larger and more complex human brain (Pollard et al., 2006).

The large number of lncRNAs that display neuronal-specific expression suggests an important role of lncRNAs in the neuronal diversification seen in higher vertebrates (Cao et al., 2006; Amaral et al., 2008; Chodroff et al., 2010; Qureshi et al., 2010). Additionally, the spatially and temporally restricted expression patterns of many lncRNAs indicate that their expression is tightly regulated, suggesting that lncRNAs may control the specification and function of individual neuronal subtypes (Mercer et al., 2008). While functional characterization of neuronal-enriched lncRNAs is still limited, broader studies of lncRNA function have implicated lncRNAs as regulators of transcription through both epigenetic regulations of chromatin structure and RNA-transcription factor interactions. Here we focus on reviewing recent advances in the identification and functional analysis of lncRNAs implicated in transcriptional regulation control of neural development.

## MECHANISMS OF lncRNA-DEPENDENT TRANSCRIPTIONAL REGULATION

In general, lncRNAs function either in *cis*, within the same genomic locus, or in *trans*, affecting gene transcription in a different locus or even on different chromosomes. Many lncRNAs, including the intensely studied *Xist* and *HOTAIR* ncRNAs, function through recruitment of the Polycomb repressive complex 2 (PRC2) by binding to PRC2 component histone-lysine N-methyltransferase Ezh2, leading to a local increase in H3K27me3 content and subsequent transcriptional repression (Zhao et al., 2008, 2010; Tsai et al., 2010; Guil and Esteller, 2012). However, other lncRNAs, like the *BORDERLINE* lncRNA, are shown to inhibit repressive histone modifications either solely through their transcription or by binding to and removing the heterochromatin protein 1 (HP1/Swi6) from the locus (Keller et al., 2013). The diverse functions observed for the handful of characterized lncRNAs studied so far underscore the importance of analyzing lncRNA function on an individual basis.

## NATURAL ANTISENSE TRANSCRIPTS

Natural antisense RNAs transcripts are lncRNAs that are transcribed from the opposite strand (OS) of protein-coding genes, and therefore, share sequence complementarity. The degree of complementarity of NATs with corresponding sense transcripts varies greatly, however, genome-wide analysis suggests that localization of antisense transcription is generally confined to 250 bp upstream of the sense transcript’s transcription start site and 1.5 kb downstream of the sense gene (Sun et al., 2005; Core et al., 2008; Seila et al., 2008). As previously reviewed, NATs mediate their function through transcriptional and epigenetic regulation, RNA–DNA interactions, and RNA–RNA interactions (Faghihi and Wahlestedt, 2009; Magistri et al., 2012). While there are clear examples of antisense transcripts that directly inhibit protein coding gene expression (Werner et al., 2014), the inhibition is probably not mediated by complementary base-pairing of sense–antisense transcripts. Since most lncRNAs are expressed at much lower levels than neighboring protein-coding genes, the stoichiometry between sense–antisense pairs is insufficient to simply block splicing or translation of protein coding genes.

One topic of particular current interest is the role of NATs that work in conjunction with epigenetic modifiers. Many imprinted genes are found in genomic clusters and have NATs located within the same locus (Verona et al., 2003; Katayama et al., 2005; Wan and Bartolomei, 2008; Mohammad et al., 2009). The imprinted locus is facilitated through allele specific expression of NATs and corresponding interactions with epigenetic modifiers. For example, the NAT *Air* interacts with HMT G9a while *Kcnq1ot1* interacts with PRC2 components and HMT G9a (Nagano et al., 2008; Pandey et al., 2008; Terranova et al., 2008). Through complementary base pairing and RNA–protein interactions, the NAT transcript allows sequence-specific recruitment of chromatin modifiers to the locus. For both *Air* and *Kcnq1ot1*, NAT expression from the paternal allele corresponds to paternal allele silencing through chromatin condensation and bidirectional spreading of epigenetic marks (Nagano et al., 2008; Kanduri, 2011). Epigenetic control of protein coding genes by NATs is also observed in non-imprinted loci. For example, brain-derived neurotrophic factor (BDNF) is regulated by the NAT *BDNF-AS*. Loss of *BDNF-AS* is accompanied by increased *BDNF* transcript abundance, facilitated through an altered chromatin state (Modarresi et al., 2012).

## INTRONIC ncRNAs

While reports suggest that up to 35% of lncRNAs localize to intronic sequences, little is known about the function of these sequences (St Laurent et al., 2012). Surprisingly, intronic ncRNAs are predominantly associated with the sense strand of the unprocessed mRNA, but often show expression patterns that are inversely correlated with the processed mRNA (Katayama et al., 2005; Nakaya et al., 2007; Dinger et al., 2008; Mercer et al., 2008). This suggests a complex regulatory relationship in which intronic ncRNA transcription may be independent of transcription of the protein coding pre-mRNA. In some cases, these intronic ncRNAs are precursor transcripts to miRNAs. Recent work has also suggested that many intron-derived RNAs bind to Ezh2 of the PRC2 complex, thus recruiting chromatin structure modifiers to the locus to silence transcription (Guil and Esteller, 2012; Guil et al., 2012).

## NON-CODING OPPOSITE-STRAND TRANSCRIPTS (ncOSTs), PROMOTER-ASSOCIATED lncRNAs, ENHANCER-ASSOCIATED RNAs (eRNAs), ULTRACONSERVED ELEMENT-ASSOCIATED lncRNAs, AND CIRCULAR RNAs

Some lncRNAs are transcribed from the proximal promoters in the opposite direction of protein coding genes, and have been termed “opposite strand” transcripts. Conservative estimates suggest that one-third of brain-enriched transcription factors express corresponding *OS* transcripts and that many of these may act in *cis* to regulate protein-coding gene transcription (Alfano et al., 2005; Rapicavoli et al., 2010). Many *OS* transcripts display correlated expression patterns with neighboring protein-coding genes as a result of bi-directional promoters initiating transcription of both the lncRNA and protein-coding gene (Uesaka et al., 2014). Recent reports analyzing the function of *Six3OS* and *Vax2OS*, however, indicate that some *OS* transcripts function in *trans*, and not by regulating expression of their neighboring protein-coding gene (Rapicavoli et al., 2011; Meola et al., 2012).

Other promoter-associated lncRNAs overlap proximal promoter sequences but are transcribed from the sense strand relative to the protein-coding gene. The transcription of the lncRNA itself can positively impact transcription in *cis* of the protein-coding gene, by changing chromatin conformation to permit transcription factor recruitment, leading to initiation of protein-coding gene transcription. Alternatively, promoter-associated lncRNAs can inhibit protein-coding gene transcription through one of two different proposed mechanisms. Chromatin de-condensation that occurs as a result of transcription of a lncRNA within the promoter region of a protein-coding gene may inhibit transcription of nearby genes by altering DNA supercoiling. Conversely, it was recently shown that transcription of the *CCND1* promoter-associated lncRNA (*CCND1-pncRNA*) recruits the TLS protein to the promoter of CCND1 during DNA damage. The recruitment of TLS reduces transcription of *CCND1* by inhibiting the histone acetyltransferase activity of CBP/p300 at the gene’s promoter (Wang et al., 2008; Kurokawa, 2011). This further suggests that some promoter-associated lncRNAs may regulate transcription of neighboring protein-coding genes through recruitment of chromatin-modifying complexes.

Past efforts in comparative genetics have identified thousands of sequences that display high sequence constraints across evolution (Bejerano et al., 2004; Woolfe et al., 2005; Pennacchio et al., 2006). These ultra-conserved regions (UCRs) identified from *Fugu rubripes* and human (Woolfe et al., 2005; Pennacchio et al., 2006) or human, mouse, and rat (Bejerano et al., 2004) are at least 200 bp and display >90% sequence conservation. The UCRs tend to cluster around genes pertinent to the regulation of organism development. Therefore, the preferential localization and high-degree of sequence conservation has led to the hypothesis that these UCRs are vital to the regulation of development. Further studies analyzing these sequences have identified that many function as enhancer sequences (Woolfe et al., 2005; Pennacchio et al., 2006). However, in these studies, many UCRs also overlapped known expressed sequence tag (EST) transcripts that were rationalized as genomic contamination or incompletely spliced pre-mRNA (Bejerano et al., 2004; Woolfe et al., 2005). Roughly 240 (50%) and 84 UCRs (6%) showed evidence for transcription in the Bejerano et al. (2004) and Woolfe et al. (2005) studies, respectively. Additional work on UCRs has since confirmed that these enhancers/UCRs can indeed be transcribed into non-coding sequence.

The discovery that many enhancers or ultra-conserved elements are not only platforms for transcription factor binding but also are transcribed themselves has stimulated studies of the role played by eRNA transcription in the regulation of neighboring genes (De Santa et al., 2010; Kim et al., 2010; Licastro et al., 2010; Wang et al., 2011). Most eRNAs are short sequences, resulting from bi-directional transcription of enhancer sequences. They exhibit H3K4me1-enriched sequences and lack poly-adenylation signals. One proposed mechanism of eRNA function in transcriptional regulation is a ripple effect, a process where growth factor-induced immediate-early gene transcription triggers initiation of transcription at nearby promoters (Ebisuya et al., 2008). One could postulate that eRNAs may function in a similar manner, with transcription factors binding to enhancers, recruiting the transcriptional machinery to the enhancer to induce eRNA transcription and chromatin modifications, leading to activation of neighboring genes. To date, however, no evidence is available to suggest such a mechanism exists for eRNA-dependent regulation of transcription. The expression of eRNAs generally correlates with activation of the neighboring gene(s; Lee, 2012; Li et al., 2013b; Melo et al., 2013; Memczak et al., 2013).

Examples exist, however, in which lncRNA sequence overlaps the ultraconserved enhancer sequence of neighboring genes (Feng et al., 2006). Transcription of lncRNA sequences from ultraconserved sequences, in sharp contrast to eRNAs, may actually inhibit antisense gene transcription of neighboring targets (Bond et al., 2009). Analyses of lncRNAs that overlap ultraconserved element sequences have been shown to possess characteristics more similar to lincRNAs, and therefore, are not typically classified as eRNAs. These signatures include H3K4me3 and modification by 3′-polyadenylation. Although many of the transcribed ultra conserved elements overlap known enhancer sequences; only a minority (~4%) are transcribed bi-directionally and are unlikely to encode short RNAs (Licastro et al., 2010). Together, this provides further indication that these lncRNAs are more similar to lincRNAs than eRNAs. One possible mechanism of function for lncRNAs that overlap ultraconserved enhancer regions comes from recent studies of the lncRNA *CCATT1-L*. *CCATT1-L* is expressed from a super enhancer region 515 kb upstream of the *MYC* locus and positively regulates *MYC* transcription by facilitating chromatin interactions between the *MYC* proximal promoter and enhancer elements (Xiang et al., 2014). This suggests that lncRNAs may facilitate transcription factor recruitment to specific DNA sequences, a potential mechanism discussed in further detail below.

Circular RNAs (circRNA) define a more unconventional and less well understood class of functional ncRNAs. These unique transcripts were originally identified in plants where they function to encode subviral components (Sanger et al., 1976). In animal species, these transcripts are thought to arise from joining of 5′ and 3′ splice sites within a single exon to form the circular transcript (Nigro et al., 1991; Capel et al., 1993; Cocquerelle et al., 1993; Chao et al., 1998; Burd et al., 2010; Hansen et al., 2011; Salzman et al., 2012). Recent profiling of mouse, human, and *Caenorhabditis elegans* identified thousands of conserved circRNAs (Memczak et al., 2013). The identified circRNAs are often highly conserved, leading the authors to hypothesize that the circRNA transcripts function as molecular decoys for RNA-binding proteins and miRNAs (Memczak et al., 2013).

## LincRNAs

Other lncRNAs do not overlap with either protein coding genes or promoter or enhancer sequences. These are collectively termed long intergenic ncRNAs (lincRNAs). Analyses of the correlated expression patterns of lincRNA and transcripts of neighboring protein-coding genes imply that lincRNAs participate in similar biological processes to neighboring protein-coding genes (Luo et al., 2013). This has been interpreted that many lincRNAs may function in *cis* to regulate expression of nearby genes (Luo et al., 2013). However, this finding also raises the possibility that lincRNAs might act in *trans* to directly or indirectly regulate the activity of co-expressed protein coding genes through RNA–protein interactions.

One classical example of lincRNA function comes from studies of *HOTAIR*. *HOTAIR* is transcribed from an intergenic region in the *HOXC* locus and is involved in recruitment of chromatin modifiers to hundreds of genomic loci (Rinn et al., 2007; Tsai et al., 2010; Chu et al., 2011). Through interactions with the PRC2 and LSD1 complexes, *HOTAIR* promotes H2K27 methylation and H3K4 demethylation, respectively, resulting in the leading to gene silencing (Rinn et al., 2007; Tsai et al., 2010; Chu et al., 2011). More specifically, *HOTAIR* expression silences expression of genes from the *HOXD* locus, thereby facilitating *HOXC* locus gene expression specifying positional identity of the *HOTAIR*-expressing cells (Rinn et al., 2007). Knockout of *Hotair* in mice causes skeletal defects including homeotic transformation of vertebrae resulting from de-repression of multiple *HoxD* cluster genes, increased expression of ~30 genes from imprinted loci and loss of vertebral boundary specification during development (Li et al., 2013a).

## COMMON FUNCTIONAL PROPERTIES OF lncRNA-DEPENDENT TRANSCRIPTIONAL REGULATION

As previously stated, many lncRNAs have been proposed to function through interactions with chromatin modifiers. In fact, it is estimated that ~30% of all lincRNAs expressed in mouse ES cells interact with one or more of 11 particular chromatin modifiers (Khalil et al., 2009; Guttman et al., 2011). This has been extrapolated to suggest that interaction with chromatin regulators is the major mechanism by which lncRNAs regulate transcription. However, many of these lncRNAs display predominantly cytoplasmic expression, suggesting instead that they may have additional cellular functions. Furthermore, there is reason to suspect that the selectivity of lncRNA–chromatin modifier interactions may have been overestimated. Experiments using overexpressed, tagged lncRNAs followed by mass spectrometry do not take into account the low transcript abundance levels seen for most lncRNAs. Chromatin-modifying enzymes are likewise abundantly expressed in virtually all cell types, particularly in comparison to transcription factors. It is thus possible that weak and possibly non-physiological interactions between lncRNAs and chromatin-modifying proteins may be detected using mass spectrometry. This may include weak interactions of highly expressed proteins that have known RNA binding potential, such as PRC2 complex proteins. Furthermore, recent reports suggest that the PRC2 protein complex is quite promiscuous in its RNA binding specificity (Davidovich et al., 2013). A more systematic interrogation of potential lncRNA–protein interactions using techniques that control for the abundance of both lncRNA and protein, such as protein microarrays, will help clarify this issue.

## LncRNAs AS MOLECULAR SCAFFOLDS FOR ORGANIZING TRANSCRIPTION AND SIGNALING

**FIGURE 2:**
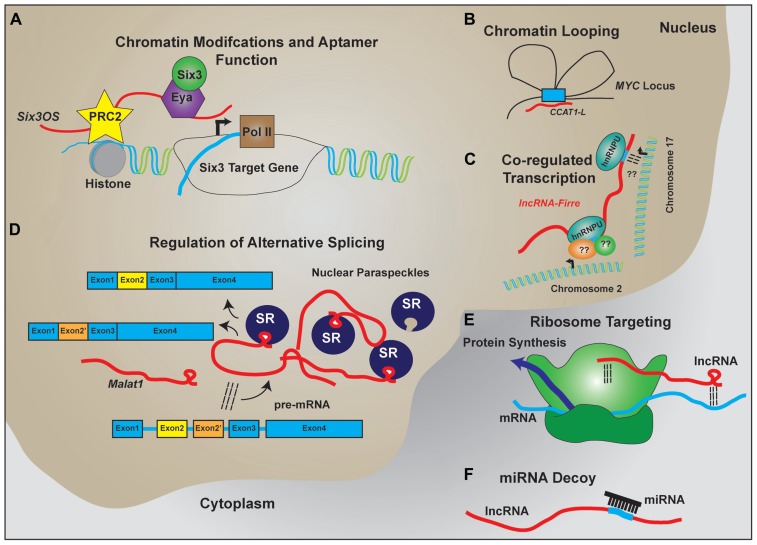
**LncRNA regulation of transcription and translation by acting as scaffolds to facilitate interactions between macromolecules**. Schematic examples of how lncRNAs participate in RNA–DNA, RNA–RNA, and RNA–protein interactions to facilitate the regulated expression of protein coding genes. **(A)** LncRNAs like *Six3OS* interact with chromatin-modifying complexes to regulate gene transcription. Additionally, lncRNAs can interact with transcription factors to facilitate target gene expression. **(B)** Complementary sequence on lncRNAs with enhancer sequences is proposed to enable chromatin looping to regulate gene transcription. **(C)** Expression of lncRNAs that contain repeat sequences for protein binding help facilitate co-regulated transcription of multiple targets, including transcription across different chromosomes. **(D)** LncRNAs are implicated in the formation and maintenance of nuclear paraspeckles that facilitate alternative splicing events of nascent transcripts. **(E)** Through homologous base-pairing with mRNA transcripts and interactions with ribosomal proteins and/or RNAs, lncRNAs are able to target mRNAs to the ribosomes. **(F)** Containing complementary target sequences, lncRNAs also serve as miRNA decoys to prevent interactions of miRNAs with protein-coding transcripts.

The characterization individual lncRNAs suggest that lncRNAs may function to serve as molecular scaffolds (**Figure 2**). Aptamer selection experiments reveal that it is relatively easy to evolve RNAs that show moderate binding affinity to a broad range of substrates, including proteins and small molecules, and demonstrate that aptamer–protein interactions show far less constraint at the level of primary sequence than do protein–protein interactions (Wilson and Szostak, 1999; Kang and Lee, 2013). In combination with homologous Watson–Crick base pairing, which provides a ready means by which RNA can selectively interact with other nucleic acid targets, this allows lncRNAs to act as molecular hubs that facilitate assembly of macromolecular complexes that can include proteins, DNA, and other RNAs.

If secondary structure primarily underlies lncRNA–protein interactions, as implied by aptamer studies, conventional sequence alignment software may not be optimal for identifying functional lncRNAs. Indeed, recent reports suggest that >20% of the human RNAs display evolutionarily conserved secondary structures independent of primary sequences (Smith et al., 2013). Reports analyzing interactions of the lncRNA *Xist, RepA*, or other short ncRNAs suggest that a double stem–loop structure is sufficient for PRC2 binding (Zhao et al., 2008; Kanhere et al., 2010). The presence of short repeats within lncRNAs that display conserved secondary structure can then facilitate protein recruitment to the regions where the lncRNA is localized. This has been recently exemplified by the lncRNA *Firre*, which contains repeat domains for nuclear matrix factor hnRNPU binding (Hacisuleyman et al., 2014). In serving as a scaffold, *Firre* is thought to mediate intra-chromosomal bridging and focalized transcription of *Firre*-regulated targets (Hacisuleyman et al., 2014).

Further evidence of lncRNAs serving as molecular scaffolds comes from studies analyzing lncRNA co-localization with the nuclear paraspeckles, domains that are thought to be locations of retained RNAs where alternative splicing events are regulated (reviewed in Spector and Lamond, 2011). The highly expressed nuclear lncRNAs *Neat1* and *Malat1* both localize to these nuclear subdomains (Clemson et al., 2009; Tripathi et al., 2010). The paraspeckle domains are thought to be locations of retained RNAs where alternative splicing events are regulated (reviewed in Spector and Lamond, 2011). *Neat1* induces paraspeckle formation and *Malat1* recruits splicing factors to these domains (Clemson et al., 2009; Tripathi et al., 2010). Through both RNA–RNA interactions and RNA–protein interactions, these lncRNAs are thus implicated in regulating splicing.

Analysis of the lncRNA *Hotair* suggests that lncRNAs can also regulate post-transcriptional processes. *Hotair* associates with the RNA-binding and ubiquitin ligase proteins Dzip3 and Mex3b (Yoon et al., 2013). Additionally, *Hotair* binds the ubiquitin ligase substrates Ataxin-1 and Snurportin-1, thereby facilitating interaction of the proteins and ubiquitin-dependent degradation of Ataxin-1 and Snurportin-1 (Yoon et al., 2013). Additional studies like these are required to address the functions of the multitude of lncRNAs that are expressed in the cytoplasm and that do not directly regulate chromatin modifications and gene transcription (van Heesch et al., 2014).

## LncRNAs IN THE DEVELOPING NERVOUS SYSTEM

Transcript expression analyses within the nervous system have shown an abundance of lncRNAs that display spatially restricted and temporally dynamic expression (Blackshaw et al., 2004; Mehler and Mattick, 2007; Mercer et al., 2008; Aprea et al., 2013; Luo et al., 2013; Lv et al., 2013). In fact, lncRNAs generally display more tissue specificity than protein-coding genes (Luo et al., 2013). The spatial and temporal regulation of lncRNAs is therefore hypothesized to promote neuronal diversification and specification. Indeed, comparative analyses of sequences from human and chimpanzee brains identified non-coding HARs that display fast evolution and are correlated with human-specific brain functions (Pollard et al., 2006). The HARs and many other lncRNAs display preferential genomic localization near protein-coding genes involved in neurodevelopment and are proposed to function through *cis-*regulation of the locus (Dinger et al., 2008; Luo et al., 2013), further implicating the requirement of lncRNA function in neurodevelopment. In addition, biological significance of lncRNAs in the developing nervous system is beginning to be understood through both loss- and gain-of-function experiments analyzing individual lncRNAs. Information regarding the identity and function of lncRNAs expressed in the developing central nervous system (CNS) is summarized in **Table 1**.

**Table 1:** LncRNAs in neurodevelopment and neurodevelopmental disorders.

lncRNA	Classification	Function	References
*AK055040*	Promoter-associated	Located upstream of CACN2D1; interacts with SUZ12 and is required for neural induction of ES cells	Ng et al. (2012)
*AK091713*	Overlapping	Contains miRNAs Mir125B and LET7A and the nuclear encoded mitochondrial protein BLID within its introns; required for neural induction of ES cells by regulated expression of miRNAs that promote neurogenesis	Ng et al. (2012)
*AK124684*	lincRNA	Interacts with the master negative regulator of neurogenesis, REST; required for neural induction of ES cells	Ng et al. (2012)
*ANRIL/*CDKN2B-AS	Antisense	CDKN2B located on the opposite strand within *ANRIL* intron1; binds chromatin-modifying complexes to regulate *CDKN2A/B* expression; mutations in promoter and transcript sequence correlate with multiple genetic disorders	Yap et al. (2010), Aguilo et al. (2011), Kotake et al. (2011), Pasmant et al. (2011a,b), Congrains et al. (2013)
*BACE1-AS*	NAT	Positive regulator of *BACE1* expression; increased expression correlates with Alzheimer’s disease pathology	Faghihi et al. (2008),
			Modarresi et al. (2011)
*BDNF-AS*	NAT	Negative regulation of BDNF through recruitment of chromatin-modifying complex to BDNF locus	Modarresi et al. (2012)
*CDRas1*	circRNA	miR-7 decoy; over-expression results in the reduced size of the zebrafish midbrain, similar to miR-7 loss-of-function	Memczak et al. (2013)
*Cyrano*	lincRNA	Loss of function results in small eyes and brains due to a reduction in neural specification; may function as a miR-7 decoy transcript	Ulitsky et al. (2011)
*Dlx1AS*	Enhancer-associated and NAT	Overlaps conserved enhancer between Dlx1/2; inhibits Dlx1 expression; loss causes increased interneuron number; required for neuronal differentiation of ES cells	Dinger et al. (2008), Mercer et al. (2010), Kraus et al. (2013)
*Evf2/Dlx6AS*	Enhancer-associated	Overlaps conserved enhancer between Dlx5/6; recruits Dlx1/2 and MECP2 to Dlx5/6 enhancer to control GABAergic interneuron specification; loss causes reduced GABAergic neuron number and reduced inhibition of CA1 pyramidal neurons	Feng et al. (2006), Bond et al. (2009), Berghoff et al. (2013)
*GDNF-OS*	Promoter-associated opposite strand	Negative regulation of GDNF	Airavaara et al. (2011), Modarresi et al. (2012)
*Gomafu/*Miat/RNCR2	lincRNA	Interacts with splicing factors to regulate alternative splicing; inhibits amacrine cell specification; associated in GWAS studies with eye movement disorders in schizophrenia; down-regulated in schizophrenic brains	Takahashi et al. (2003), Rapicavoli et al. (2010), Tsuiji et al. (2011), Barry et al. (2013)
*Kcna2AS*	NAT	Inhibits expression of *Kcna2*; results in decreased voltage-gated potassium currents and increased resting membrane potential leading to pain hypersensitivity	Zhao et al. (2013)
*Linc-Brn1b*	lincRNA	Loss causes reduced *Brn1* expression, reduced proliferation of intermediate progenitors in the SVZ of the dorsal telencephalon and fewer upper layer cortical neurons	Sauvageau et al. (2013)
*Linc00299*	lincRNA	Deletion/interruption results in cognitive developmental delay in humans caused by improper neural development	Talkowski et al. (2012)
*Malat1*	lincRNA	Regulation of alternative splicing through recruitment of slicing factors to paraspeckles; regulation of synaptogenesis through gene splicing; *cis-* regulation of gene expression	Bernard et al. (2010), Tripathi et al. (2010), Zong et al. (2011), Zhang et al. (2012)
*Megamind*	lincRNA	Loss of function results in small eyes and brains due to a reduction in neural specification	Ulitsky et al. (2011)
*Neat1*	lincRNA	Induction of paraspeckle formation	Clemson et al. (2009)
*Paupar*	lincRNA; Promoter-associated?	Negative regulation of Pax6 expression; regulation of Pax6 target gene (and others) expression through occupancy of promoter sequences	Vance et al. (2014)
*RMST*	lincRNA	Binds to Sox2 and the promoters of Sox2 targets to facilitate Sox2-dependent neural induction	Ng et al. (2012, 2013)
*Six3OS*	Promoter-associated opposite strand	Regulation of Six3 targets through interactions with Eya proteins and the chromatin-modifying protein Ezh2; required for neural specification of ES cells	Alfano et al. (2005), Zhao et al. (2010), Rapicavoli et al. (2011)
*Sox2dot*	Enhancer-associated	Overlaps a distal enhancer of Sox2; expressed in neurogenic regions of the brain	Amaral et al. (2009)
*Tug1*	lincRNA	Up-regulated in response to taurine; inhibition of cone photoreceptor specification through PRC2 complex-mediated chromatin modifications affecting cell-cycle regulation	Altshuler et al. (1993), Young et al. (2005), Khalil et al. (2009)
*TUNA*	lincRNA	Regulates pluripotency by recruiting RNA biding proteins to Sox2, Nanog, and Fgf4 promoters; required for neural specification of ES cells	Lin et al. (2014)
*utNgn1*	Enhancer-associated	Located ~6 kb upstream of *Neurog1*gene; required for proper *Neurog1* transcription and mouse cortical progenitor differentiation	Onoguchi et al. (2012)
*Vax2OS*	Promoter-associated opposite strand	Maintenance of proliferation through alterations to cell cycle progression in neural progenitors	Meola et al. (2012)

### INSIGHTS FROM CONTROLLED DIFFERENTIATION OF ES CELLS

Recent studies have focused on the identification of lncRNAs expressed during neuronal differentiation, either in stem cells or *in vivo.* The rationale behind these studies suggests that the identification of lncRNAs that display dynamic expression across developmental stages can be extrapolated to lncRNA participation in differentiation. For example, expression profiling of embryoid body (EB) differentiation of mouse embryonic stem cells (ES) revealed 174 lncRNAs that displayed differential expression patterns (Dinger et al., 2008). Consistent with previous reports on protein-coding gene expression in pluripotent cells (Ivanova et al., 2002; Ramalho-Santos et al., 2002; Bruce et al., 2007; Dinger et al., 2008), twice as many lncRNAs were expressed during pluripotent stages versus more committed lineages (Dinger et al., 2008). Overall, 12, 7, and 31 lncRNAs displayed dynamic expression patterns consistent with pluripotency, primitive streak formation/gastrulation and hematopoiesis, respectively, with many lncRNAs displaying expression patterns with positive correlations to neighboring protein-coding genes (Dinger et al., 2008). Further reports have identified 226 lncRNAs expressed in pluripotent ES cells (Guttman et al., 2009, 2010), 137 of which were knocked-down and showed a significant impact on ES cell gene expression (Guttman et al., 2011). Importantly, loss-of-function studies indicated that 26 of these lncRNAs function to maintain ES cell pluripotency (Guttman et al., 2011). In both studies, many identified lncRNAs were proposed to regulate gene transcription through identified RNA–protein interactions of lncRNA and protein components of chromatin-modifying complexes (Dinger et al., 2008; Guttman et al., 2011). The importance of lncRNAs in pluripotency was further confirmed through observations where two lncRNAs, themselves transcriptional targets of Oct4 and Nanog, regulate pluripotency through a feedback-loop regulating *Oct4* and *Nanog* transcript expression (Sheik Mohamed et al., 2010).

Additional studies have more specifically characterized the requirement of lncRNAs in neural and oligodendrocyte induction (Mercer et al., 2010; Ng et al., 2012). In comparing neural progenitor cells differentiated from human ES cells, Ng et al. (2012) observed 934 of 6671 lncRNAs that displayed differential expression by microarray analysis. Similar to previous reports in mouse ES cells, 36 lncRNAs displayed expression patterns consistent with regulation of pluripotency, three of which were experimentally shown to regulate pluripotency through knockdown studies and contained OCT4- and NANOG-binding sites in their proximal promoter. Further characterization through RNA immunoprecipitation (RIP) indicated that two lncRNAs interacted directly with the pluripotency transcription factor SOX2 and the PRC2 chromatin-modifying complex component SUZ12 (Ng et al., 2012). In these studies, 35 lncRNAs displayed expression patterns consistent with a role in neural induction, four of which were studied and shown to be required for proper neural differentiation. Of these four lncRNAs, one (AK055040) was shown to interact with SUZ12, indicating a functional role in chromatin modifications in the regulation of neurogenesis. An additional lncRNA (AK124684) was found to interact with the transcriptional factor REST (Ng et al., 2012), a master negative regulator of neurogenesis that binds to the promoters of neurogenic genes to inhibit gene transcription (Ballas et al., 2005; Abrajano et al., 2009; Gao et al., 2011). A third lncRNA (AK091713) was subsequently shown to contain miRNAs miR-125b and let-7a within its intronic sequence, thereby driving neurogenesis through the expression of neurogenic miRNAs (Rybak et al., 2008; Le et al., 2009; Ng et al., 2012). Other studies identified that the lncRNAs *Six3OS* and *Dlx1AS* are required for directed differentiation of pluripotent stem cells towards a neuronal precursor identity (Ramos et al., 2013).

Among lncRNAs found to regulate neurogenesis, the lincRNA *RMST* was targeted for additional follow-up studies. *RMST* in humans is located ~150 kb away from the closest annotated protein-coding gene (Ng et al., 2012). The promoter of *RMST* contains REST binding sites and is occupied by REST, suggesting that *RMST* is activated during neurogenesis through dissociation of REST from the promoter (Ng et al., 2013). Analysis of *RMST* revealed that *RMST* promotes neurogenesis through inhibition of glial fates (Ng et al., 2013). RNA pull-down experiments indicated that *RMST* interacts with the RNA binding protein hnRNPA2/B1 and SOX2, both of which are also required for neuronal differentiation (Ng et al., 2013). Ultimately, it was observed that *RMST* regulates neuronal differentiation through directing SOX2 to the promoter of neurogenic transcription factors, to promote neurogenic gene expression and neural fate commitment (Ng et al., 2013). *RMST* does not bind REST or the PRC2 chromatin-modifying complex protein SUZ12 (Ng et al., 2012). Using both RIP and chromatin isolation by RNA purification (ChiRP) to identify DNA-binding sites of lncRNAs (Chu et al., 2011), the researchers provided evidence that *RMST* binds to promoters of Sox2 target genes, and activates transcription of these genes by recruiting Sox2 (Ng et al., 2013). The mechanism by which *RMST* is recruited to Sox2 consensus binding sites is unclear, but is postulated to occur through homologous base pairing that leads to the formation of RNA–DNA hybrids (Ng et al., 2013). If this is the case, this may turn out to be a more general mechanism by which *trans*-acting lncRNAs regulate gene expression.

Similar to *RMST*, *utNgn1*, and *Sox2dot* display expression profiles that positively correlate with differentiation of neural progenitors (Amaral et al., 2009; Onoguchi et al., 2012). Importantly, both of these lncRNAs overlap sequences of ultra conserved elements implicated in neuronal development (Amaral et al., 2009; Onoguchi et al., 2012). *UtNgn1* is required for *Neurogenin1* (*Neurog1*) transcription and PRC2-mediated repressive signals at the *utNgn1* locus are associated with both decreases in *utNgn1* and *Neurog1* transcript abundance (Onoguchi et al., 2012). Inhibition of *utNgn1* expression during mouse cortical progenitor differentiation resulted in decreased expression of neurogenic markers, consistent with a role of *utNgn1* in promoting neurogenesis through activation of *Neurog1* transcription (Onoguchi et al., 2012). The exact mechanism by which transcription of *utNgn1* at the Neurog1 enhancer mediates *Neurog1* transcription remains elusive. Similarly, expression of *Sox2dot* in the neurogenic regions of the brain suggests that it functions to regulate neural development (Amaral et al., 2009). The function of *Sox2dot* in neural development remains to be investigated.

More recent experiments have identified 20 additional lncRNAs that regulate pluripotency. In particular, the lncRNA *TUNA*, was shown to be highly conserved among vertebrates and was expressed within the developing nervous system (Lin et al., 2014). *TUNA* was shown to regulate pluripotency through the binding of three RNA binding proteins and co-occupancy of the RNA–protein complex at the promoters of *Sox2*, *Nanog*, and *Fgf4*. Inhibition of *TUNA* resulted in the decreased capacity of mESCs to differentiate to neural lineages (Lin et al., 2014). Consistent with a role in regulating neural development, *TUNA* expression is correlated with Huntington’s disease (HD) prognosis, and inhibition of *TUNA* in zebrafish results in locomotor defects (Lin et al., 2014).

### CONTROL OF NEURAL DEVELOPMENT *IN VIVO* BY lncRNAs

#### LncRNAs in retinal development

While the identification and validation of the function of lncRNAs during neuroectodermal differentiation from cultured ES cells have provided a wealth of information regarding which lncRNAs to target, relatively few studies have begun to examine the role of individual lncRNAs *in vivo* during neurodevelopment. To date, many examples of lncRNA function *in vivo* come from studies analyzing the role of ncRNAs during retinal development (reviewed in Rapicavoli and Blackshaw, 2009). Specifically, four lncRNAs have been implicated in regulating cell fate decisions during retinal development. *Tug1* was identified in a screen to characterize genes that display enhanced expression in response to taurine, which promotes rod photoreceptor differentiation (Altshuler et al., 1993; Young et al., 2005). *Tug1* knock-down experiments displayed abnormal morphology of inner and outer segments of photoreceptors, accompanied by increased cell death and an increase in the percentage of electroporated cells expressing the cone-photoreceptor marker peanut agglutinin (PNA; Young et al., 2005). Studies analyzing the interactions of lincRNAs with chromatin-modifying complexes identified an association between *TUG1* and the PRC2 complex (Khalil et al., 2009). Further characterization of *Tug1* revealed that it is activated in a p53-dependent manner, and loss of *Tug1* results in the up-regulation of ~120 genes, most of which are genes involved in cell-cycle regulation (Guttman et al., 2009; Khalil et al., 2009). Combined, these results indicated that *Tug1* functions to promote rod genesis through inhibition of cone photoreceptor cell fate through its interactions with repressed chromatin (Young et al., 2005; Khalil et al., 2009). However, only a subset of cellular *Tug1* RNA is localized to the nucleus, suggesting that other mechanisms of *TUG1* function may exist (Khalil et al., 2009).

The lncRNA*Vax2os* has been shown to display predominately retinal expression, specifically at post-natal periods during mouse development (Alfano et al., 2005). *Vax2os1* was also found to regulate mouse photoreceptor differentiation (Meola et al., 2012). *Vax2os1* is endogenously expressed in the ventral retina of mice, primarily localizing to the outer neuroblastic layer of the developing retina. Overexpression of *Vax2os1* increases the proportions of proliferating cells in the dorsal retina (low endogenous expression of *Vax2os1*) through perturbation of cell cycle progression in neural progenitors (Meola et al., 2012). The increase in proliferative progenitors and increased apoptosis in *Vax2os1* overexpressing cells resulted in a decrease of photoreceptor differentiation (Meola et al., 2012).

The lncOST *Six3os* is co-expressed with the homeodomain transcription factor *Six3* in retinal progenitor cells (Blackshaw et al., 2004). *Six3os* is juxtaposed to *Six3* and transcribed in the opposite direction of *Six3* in both mouse and human (Alfano et al., 2005; Rapicavoli et al., 2011). *Six3os*, however, does not regulate *Six3* transcription. The *Six3os* transcript forms an RNA–protein complex with transcriptional co-regulators of Six3 such as Eya1, but not with Six3 itself, suggesting that *Six3os* controls expression of Six3 target genes (Alfano et al., 2005; Rapicavoli et al., 2011). Furthermore, *Six3os* interacts with the Ezh2 component of the PRC2 complex (Zhao et al., 2010; Rapicavoli et al., 2011), suggesting that *Six3os* may function to repress Six3 targets by triggering H3K27me3 modification. This is further supported by experiments in which *Six3os* overexpression blocked changes in retinal cell fate induced by *Six3* overexpression (Rapicavoli et al., 2011). Inhibition of *Six3os* expression resulted in a decrease in rod bipolar cells with a concomitant increase in Müller glial cell number (Rapicavoli et al., 2011). This phenotype is similar to loss of function of *Six3* alone (Zhu et al., 2002; Rapicavoli et al., 2011).

Another lncRNA that is also prominently expressed in the retina and has recently been functionally characterized is *Gomafu* (also known as *RNCR2* and *Miat*). *Gomafu* is one of the most abundant polyadenylated RNAs found in the neonatal retina (Blackshaw et al., 2004), and is expressed widely throughout the nervous system, displaying nuclear localization to a novel nuclear domain within neural precursors (Ishii et al., 2006; Sone et al., 2007; Chen and Carmichael, 2010). Overexpression of *Gomafu* in the developing retina had no effect on retinal development, presumably due to the already high abundance levels of *Gomafu* transcript (Rapicavoli et al., 2010). Inhibition of *Gomafu* expression/function resulted in an increase in amacrine and Müller glial cells in the developing retina, suggesting that *Gomafu* negatively regulates amacrine cell fate specification and delays Müller glial cell specification (Rapicavoli et al., 2010). Additional studies on *Gomafu* revealed that it selectively bound splicing regulators such as SF1 and Qk, and that its loss of function disrupted splicing of a subset of neuronal pre-mRNAs (Tsuiji et al., 2011; Barry et al., 2013). However, the mechanism by which *Gomafu*-dependent mRNA splicing affects amacrine and Müller glial cell specification remains elusive. Since many other lncRNAs are prominently expressed in the developing retina (Blackshaw et al., 2004), further studies will undoubtedly identify further instances in which lncRNAs regulate the expression and/or activity of protein-coding genes essential for retinal development.

#### LncRNAs that regulate development of other CNS regions

Although the study of lncRNAs in other regions of the developing CNS has lagged behind studies in retina until recently, this is now rapidly changing. At least a half-dozen lncRNAs have now been functionally characterized in developing brain. Several examples of functional lncRNAs have been identified through analysis of the transcriptional control of GABAergic interneuron specification. During development, multipotent progenitors that give rise to both GABAergic interneurons and oligodendrocytes are generated from the medial and caudal ganglionic eminences of the ventral telencephalon (Anderson et al., 1997; Panganiban and Rubenstein, 2002; Yung et al., 2002). *In vitro* differentiation of embryonic forebrain-derived neural stem cells identified a host of additional lncRNAs dynamically expressed during GABAergic interneuron specification (Mercer et al., 2010), including two lncRNAs that overlap ultraconserved enhancers of the DLX family of proteins, *Dlx1AS* and *Evf2*.

*Evf2* is partially transcribed from an ultra-conserved enhancer sequence (ei) located between the convergently transcribed *Dlx5* and *Dlx6* genes (Feng et al., 2006). *Evf2* is transcribed in the antisense direction to Dlx6, with the entire sequence for *Dlx6* localized within intron 2 of *Evf2* (Feng et al., 2006). Transcription of *Evf2* results in the recruitment of Dlx1/2 and MECP2 transcription factors to the *Dlx5/6* enhancers to regulate *Dlx5/6* transcription (Feng et al., 2006; Bond et al., 2009). Loss of *Evf2* results in an increase in *Dlx6* transcript abundance, a phenotype that cannot be rescued with *Evf2* overexpression, suggesting that transcription of *Evf2* inhibits activation of *Dlx6* transcription in *cis* through opposite-strand inhibition (Bond et al., 2009). Further studies indicate that *Evf2 trans* activity inhibits the ei enhancer methylation (Berghoff et al., 2013). Altogether, *Evf2* functions in both *cis* and *trans* to regulate transcription of Dlx5/6 and chromatin status of the ei ultra-conserved enhancer.

*Dlx1AS* is localized in the *Dlx1/2* locus similar to *Evf2* in the *Dlx5/6* locus, such that *Dlx1AS* overlaps the conserved enhancer between the convergently transcribed *Dlx1/2* genes (Dinger et al., 2008). In contrast to the genomic architecture of *Evf2*, exon 2 of *Dlx1AS* overlaps the *Dlx1* coding sequence in the antisense orientation, suggesting *Dlx1AS* may also function as a NAT (Kraus et al., 2013). Genetic loss of *Dlx1AS* results in increased Dlx1 expression, suggesting a negative regulation of *Dlx1* by *Dlx1AS*, potentially through antisense inhibition (Kraus et al., 2013). These reports suggest that lncRNAs transcribed from ultra conserved sequences can function through molecular mechanisms shared with other classes of lncRNAs. They may also control activity and/or recruitment of transcription factors at enhancers through dosage or allelic differences in lncRNA abundance, adding an additional layer of complexity to enhancer-mediated gene regulation (Amaral et al., 2009).

In order to study loss of function of *Dlx1AS* and *Evf2 in vivo*, homologous recombination was used to insert premature poly-adenylation sequences in both lncRNAs, as genomic deletion of either *Dlx1AS* or *Evf2* would alter expression or affect primary sequence of neighboring protein-coding genes (Bond et al., 2009; Kraus et al., 2013).

Insertion of the transcriptional terminator sequence in the *Evf2* locus results in a significant, but incomplete loss of lncRNA transcript expression, likely resulting in a hypomorphic phenotype. Loss of *Evf2* results in an early decrease in GABAergic neurons in the hippocampus and dentate gyrus in juvenile mice (Bond et al., 2009). Although the deficit in GABAergic neuron number is recovered in in adult mice, loss of *Evf2* results in reduced inhibition of CA1 pyramidal neurons, likely the result of synaptic defects from reduced Gad1 levels (Bond et al., 2009).

Similarly, in addition to mild defects resulting in craniofacial anomalies, loss of *Dlx1AS* also affects the number of hippocampal interneurons (Kraus et al., 2013). Loss of *Dlx1AS* results in increased interneuron number, likely due to an increase in *Dlx1* expression that triggers a corresponding increase of expression of *Mash1* (Kraus et al., 2013). Similar to *Evf2* studies, early changes in interneuron number are not maintained into adulthood in *Dlx1AS* mice, suggesting compensatory mechanisms regulating proper number of neurons (Kraus et al., 2013). Combined with the observations of decreased *Olig2* expression in *Dlx1AS* mutant mice, *Evf2* and *Dlx1AS* may be functioning to control levels of the Dlx protein family to generate the proper proportion of oligodendrocytes and GABAergic neurons generated from the bipotent precursor (Bond et al., 2009; Kraus et al., 2013). Other studies have indicated that lncRNAs can play a pivotal role in controlling neural versus oligodendrocyte fate decisions. This includes studies in which *Nkx2.2AS* was overexpressed in ventral telencephalic progenitors and was observed to drive oligodendrocyte specification, possibly by increasing Nkx2.2 levels (Tochitani and Hayashizaki, 2008).

Studies in zebrafish have examined conserved lincRNAs that display short sequences of high homology across evolution and syntenic genomic localization (Ulitsky et al., 2011). The lncRNAs *cyrano* and *megamind* are highly expressed throughout the developing nervous system. Morpholino knockdown of *cyrano* and *megamind* results in zebrafish with reduced brain and eye size (Ulitsky et al., 2011). Additional phenotypes include neural tube closing defects and reduced accumulation of the NeuroD-GFP positive neurons in the developing eyes and brain (Ulitsky et al., 2011). In examining the evolutionary conservation of function of lncRNAs, the researchers showed that the syntenic mouse and human lncRNAs could partially rescue the observed phenotypes from *megamind* inhibition. Additionally, the rescue using mouse and human orthologs was dependent on expression of the evolutionarily conserved sequence (Ulitsky et al., 2011). Interestingly, the conserved sequence of *cyrano* was not sufficient to rescue decreased *cyrano* expression (Ulitsky et al., 2011). The conserved sequence of *cyrano*, however, matched the consensus binding sequence of miR-7, suggesting regulation of *cyrano* by miR-7, or conversely, *cyrano* functioning as a miRNA decoy (Ulitsky et al., 2011).

Similar to *cyrano*, the circRNA *CDR1as* also serves as a miR-7 decoy. *CDR1as* is highly conserved amongst mammals and contains 63-consensus miR-7 binding sites conserved among two or more species (Memczak et al., 2013). *CDR1as* is an antisense transcript to the *CDR1* coding sequence and shares a similar expression pattern to miR-7 during brain development. Over-expression of the human *CDR1as* in zebrafish, which have lost the entire CDR1 locus, results in a decreased size of the midbrain, similar to miR-7 loss-of-function (Memczak et al., 2013). Together, these data suggest that *CDR1as* acts as an endogenous “sponge” that attenuates the action of miR-7 on protein coding mRNAs through competitive binding.

Like *Six3os* in retina, recent experiments examining the lncRNA *Paupar* have uncovered another instance of a lncRNA that cooperates with the neighboring protein-coding gene to regulate transcription (Vance et al., 2014). *Paurpar* is localized ~8.5 kb upstream of the homeodomain factor *Pax6*, which regulates many different aspects of CNS development. Interestingly, *Paupar* is localized within the first intron of the ncRNA *Pax6os1*, and is generally coexpressed with *Pax6* mRNA. However, *Paupar* inhibition results in an increase in *Pax6* expression. Comparing changes in gene expression seen following knockdown of *Paupar* and *Pax6* revealed many genes that showed similar changes in expression, indicating that while *Paupar* regulates expression of *Pax6* itself, *Paupar* is also likely to participate in the regulation of Pax6 target genes (Vance et al., 2014). Using capture hybridization analysis of RNA targets (CHART), the researchers found that *Paupar* occupied >2500 genomic sites, localizing to the promoters of many genes involved in stem cell maintenance and neuronal development (Vance et al., 2014). Further characterization indicated that Paupar and Pax6 co-occupy 71 different genomic loci, suggesting that both directly co-regulate transcription of these genes (Vance et al., 2014). It remains to be determined, however, if Paupar and Pax6 physically associate to regulate target genes. It will also be important to examine the Pax6-independent functions of *Paupar* as a majority of the genomic binding sites of Paupa*r* are not co-occupied by Pax6.

Recently, a small consortium has targeted multiple lincRNAs for genetic deletion and begun reporting phenotypic analyses (Sauvageau et al., 2013). In their studies, seven of the 18 lincRNAs targeted for knockout were shown to have human orthologs that were dynamically expressed during directed neuronal differentiation of ES cells (Sauvageau et al., 2013). In particular, the deletion of the lincRNA *linc-Brn1b* was analyzed. *Linc-Brn1b* is localized less than 10 kb downstream of the *Brn1* gene, and is transcribed from the OS of *Brn1* (Sauvageau et al., 2013). Deletion of *linc-Brn1b* results in mice with reduced *Brn1* transcript abundance. These mutants display features similar to Brn1/Brn2 double knockouts, including reduced proliferation of intermediate progenitors within the sub-ventricular zone (SVZ) of the dorsal telencephalon, reduced production of upper layer cortical neurons and a reduction in total size of the barrel cortex (Sauvageau et al., 2013). As *linc-Brn1b* was completely excised in the knockout studies and the phenotypes mimic some features of Brn1 knockouts, the possibility exists that the observed phenotypes are partially the result of decreased *Brn1* expression due to a lost enhancer sequence within *linc-Brn1b* (Sauvageau et al., 2013). Further characterization of *linc-Brn1b* and other lincRNA knockout lines generated in these studies will continue to elicit the importance of lncRNAs in neuronal development.

## LncRNAs IN DISORDERS OF THE NERVOUS SYSTEM

Many groups are taking advantage of RNA-Seq and lncRNA microarray technologies to identify altered transcript expression levels between control and diseased states within various human neurological and psychiatric disorders (Dharap et al., 2012, 2013; Petazzi et al., 2013; Ziats and Rennert, 2013). While useful, with few exceptions, these studies have not functionally implicated these lncRNAs in disease progression (Petazzi et al., 2013; Ziats and Rennert, 2013). Here we review the limited number of studies that directly link altered lncRNA function to the development and progression of neurological disease.

One of the better studied lncRNAs associated with human disease is *ANRIL* (also known as *CDKN2B-AS*). Genome-wide association studies have associated numerous polymorphisms on human chromosome 9p21 that segregate with diseases including cardiovascular disease, Type-2 diabetes, Alzheimer’s disease (AD), primary open angle glaucoma, endometriosis, periodontitis, and several cancers (reviewed in Congrains et al., 2013). Polymorphisms map to both the promoter and transcribed region of *ANRIL*, including many transcription factor-binding sites located throughout the locus. *ANRIL* has been shown to bind CBX7 and SUZ12 of the PRC1 and PRC2 complexes, respectively, to regulate the histone modification status of the nearby *CDKN2A* and *CDKN2B* genes (Yap et al., 2010; Aguilo et al., 2011; Kotake et al., 2011). As both increased and decreased *ANRIL* expression levels correlate with disease states (Congrains et al., 2013), the fine control of *CDKN2B/CDKN2A* transcript abundance seems paramount to normal development.

*Kcna2AS* is an antisense ncRNA to the voltage-gated potassium channel Kcna2. Expression of *Kcna2AS* was observed in dorsal root ganglia (DRG) and was expressed at higher levels in ganglia exhibiting lower levels of Kcna2 protein expression, or after spinal nerve injury (Zhao et al., 2013). Spinal nerve injury causes an increase of myeloid zinc finger protein 1 (MZF1) binding to the proximal promoter of *Kcna2AS*, causing an increased expression of *Kcna2AS* with a concomitant decrease in Kcna2 transcript and protein abundance (Zhao et al., 2013). Additional experiments found that expression of *Kcna2AS* causes a decrease in voltage-gated potassium currents and an increase in membrane resting potential, suggesting that pain hypersensitivity or neuropathic pain can be caused by altered *Kcna2AS* levels (Zhao et al., 2013).

Recent studies have also implicated altered lncRNA expression as associated with AD progression. AD is characterized by a progressive neurodegeneration that leads to memory and cognitive impairment. A hallmark component of the pathological condition is the buildup of extracellular beta amyloidal plaques. The amyloid precursor protein (APP) is cleaved in the initial and rate-limiting step by β-secretase enzyme (BACE1) to form the amyloid β precursor proteins Aβ 1–40 and Aβ 1–42. In pathological conditions, the Aβ 1–42 proteins oligomerize and contribute to the plaques that participate in AD (Abramov et al., 2004; Ohyagi et al., 2005; Snyder et al., 2005; Esposito et al., 2006; Zhu et al., 2006; Lacor et al., 2007; Matsuyama et al., 2007). As a result, it has been suggested that BACE1 misregulation can contribute to excess Aβ 1–42 protein production and the development of amyloid plaques. Recent work has identified an antisense transcript to BACE1 (*BACE1-AS*) that encodes a conserved ~2 kb lncRNA with a 104 bp overlap with the human *BACE1* transcript (Faghihi et al., 2008). Both overexpression and knockdown experiments indicated that *BACE1-AS* is a positive regulator of *BACE1* transcript and protein abundance (Faghihi et al., 2008). Mechanistically, *BACE1-AS* stabilizes the *BACE1* transcript, protecting it from RNA degradation through RNA–RNA pairing of the *BACE1-AS* and *BACE1* homologous regions (Faghihi et al., 2008). Importantly, *BACE1-AS* and *BACE1* transcripts were induced by many cell stressors that are implicated in the initiation of AD, suggesting a direct mechanism by which cell stress can lead to increased Aβ precursor protein production (Faghihi et al., 2008). The importance of *BACE1-AS* in AD was further supported through examinations of primary tissues from multiple brain regions, where *BACE1-AS* transcript abundance was elevated twofold in confirmed AD patient brain samples compared to age- and sex-matched controls (Faghihi et al., 2008). Further characterization of *BACE1-AS* in a transgenic mouse model of AD indicated that *BACE1-AS* inhibition reduces the insoluble fraction of Aβ 1–40 and Aβ 1–42 precursor proteins (Modarresi et al., 2011), suggesting that increased *BACE1-AS* expression does directly contribute to AD pathology.

Other aspects of AD are also potentially regulated through lncRNA function. Recent work on neurotrophin levels in diseases of the brain have indicated that reduced neurotrophin levels (BNDF and glial derived neurotrophic factor – GDNF) correlate with the onset of neurodegenerative disorders such as Parkinson’s disease, AD, and HD (reviewed in Allen et al., 2013). This has led to potential therapeutics aimed at increasing neurotrophin levels (Weinreb et al., 2007; Straten et al., 2011; Allen et al., 2013). However, as both BDNF and GDNF display complex splicing regulation (Airavaara et al., 2011; Modarresi et al., 2012), other mechanisms of therapeutic intervention than exogenous neurotrophin replacement may be better suited to treating the diseases. Interestingly, both BDNF and GDNF have corresponding anti-sense or OS transcripts (*BDNF-AS* and *GDNF-OS*), however, one of the three *GDNF-OS* transcripts is likely protein coding (Airavaara et al., 2011). Knockdown of either *BDNF-AS* or *GNDF-OS* results in an increase in corresponding protein-coding gene transcript abundance, implying that these lncRNAs negatively regulate neurotrophin expression (Modarresi et al., 2012). Further characterization of *BDNF-AS* indicates that *BDNF-AS* recruits EZH2 and the PRC2 complex to the BDNF promoter to repress *BDNF* transcription through H3K27me3 histone modifications (Modarresi et al., 2012). Combined with studies in which treatment with exogenous BDNF rescued HD phenotypes in mice (Xie et al., 2010), these experiments suggest that inhibition of neurotrophin antisense transcripts may provide a novel target for treatment of neurodegenerative disease.

LncRNAs have also been implicated in nervous system disorders through their role in pre-mRNA splicing. The lncRNAs *Gomafu* and *Malat1* are both highly expressed in the nervous system and regulate splicing through interactions with splicing factors (Sone et al., 2007; Tripathi et al., 2010; Tsuiji et al., 2011; Zong et al., 2011). Interestingly, aberrant splicing of the genes DISC1 and ERBB4, among others, is associated with disease pathology in schizophrenia (SZ; Law et al., 2007; Nakata et al., 2009; Morikawa and Manabe, 2010). Additionally, the *Gomafu*-bound splicing factor QKI is downregulated in SZ brains and is proposed to contribute to disease pathology (Aberg et al., 2006a, b; Haroutunian et al., 2006; McCullumsmith et al., 2007). Recently, *Gomafu* has been shown to interact with multiple splicing factors, including a strong interaction with QKI (Barry et al., 2013). *Gomafu* expression is also significantly decreased shortly after neuronal depolarization in the cortical neurons in mice, and in human induced pluripotent stem cell (iPSC)-derived neurons (Barry et al., 2013). Combined with GWAS studies linking *Gomafu* with eye movement disorders in SZ (Takahashi et al., 2003), this led to the hypothesis that loss of function of *Gomafu* may directly contribute to SZ disease pathology. Indeed, *Gomafu* is significantly reduced in superior temporal gyrus of SZ brain samples compared to controls (Barry et al., 2013). Knockdown of *Gomafu* in iPSC neurons also results in an increase in rare splice variants of *DISC1* and *ERBB4* (Barry et al., 2013), matching splicing patterns observed *in vivo* from human SZ brains (Law et al., 2007; Nakata et al., 2009).

With the increased use of whole exome sequencing and copy number variations (CNV) for genetic analysis of patients with neurological diseases, our understanding of the importance of lncRNAs in neurodevelopment will only be further increased. For example, one patient that displayed a cognitive developmental delay possessed a chromosomal translocation that affected *linc00299* (Talkowski et al., 2012). Further examinations of patient databases identified an additional four patients that displayed developmental delay and disruption of the *linc00299* locus (Talkowski et al., 2012), suggesting that *linc00299* is vital for proper neuronal development. Further characterization of lncRNA function in animal models and *in vitro* will continue to expand our knowledge on the importance of lncRNAs in both human development and disease.

## CONCLUSION

Advances in sequencing technologies and the appreciation of functional non-coding elements have resulted in the rapid identification of a plethora of lncRNAs expressed in both vertebrate and invertebrates, alike. Systematic characterization of temporally and spatially restricted expression patterns in the developing nervous system has provided the groundwork for hypotheses regarding lncRNA function. As we understand more about the mechanism by which lncRNAs are regulating transcription, we are beginning to understand the biological significance of what once was labeled as “junk DNA.” Many lncRNAs regulate transcription through regulation of epigenetics and interactions with chromatin-modifying complexes, although the mechanism by which lncRNAs are recruited to specific genomic loci is still unclear. Recently developed technologies have the potential to greatly expand our understanding of the mechanism by which lncRNAs function. The advent of techniques such as ChIRP and CHART allow for systematic characterization of DNA binding sites of lncRNAs throughout the genome (Chu et al., 2011; Simon et al., 2011). Additionally, protein arrays, as used to identify *Six3os* binding partners (Rapicavoli et al., 2011), allow for an unbiased approach to identifying physiologically relevant protein binding partners. These techniques will further our understanding of how lncRNAs function as molecular scaffolds and will enable the functional characterization of lncRNAs working in *trans*. While not the focus of this review, it is also essential to consider the function of lncRNAs that display cytoplasmic expression, which represent a large fraction of lncRNAs and whose function is poorly understood (reviewed in Batista and Chang, 2013). Further characterization of lncRNA–protein interactions through protein arrays will help facilitate these discoveries. As many cytoplasmic lncRNAs associate with ribosomes (van Heesch et al., 2014), it is intriguing to speculate that lncRNAs function as scaffolds to regulate localized protein synthesis and/or degradation, a concept vitally important in the control of synaptic function.

As we continue to understand the molecular basis of lncRNA function, it is imperative that studies move from *in vitro*, homogeneous cell populations and begin to examine the consequence within individual cell types. Neuronal diversification has exhibited a multitude of examples in which transcriptional regulation and cell-fate decisions are very context and cell-type specific. Therefore, it is plausible that individual lncRNAs may display diverse functions that are dependent on their spatial and temporal expression pattern. Inherent to the examination of specific cell types is that epigenetic marks may display vast temporal and/or cell-type specific signatures. *In vivo* experiments continue to shed light on the importance of lncRNA function throughout neuronal development. As mouse models for genetic loss of lncRNAs such as *Evf2*, *Dlx1AS*, *Malat1*, and *Neat1* produce modest phenotypes or fail to recapitulate phenotypes observed in knockdown experiments (Bond et al., 2009; Zhang et al., 2012; Kraus et al., 2013), it is important to consider that lncRNAs may have evolved to function as a fine-tuning mechanism to ensure proper regulation of neuronal cell type proportions in the highly complex mammalian nervous system. Genetic compensation may mask phenotypes resulting from conventional gene knockout approaches, which conditional or acute loss of function studies may readily detect. Furthermore, efforts need to be made to carefully examine genetic models of lncRNA loss-of-function, however, being constantly mindful of the fact that many lncRNAs overlap conserved regulatory elements that may have function independent of the lncRNA itself, complicating interpretation of any observed phenotypes. Further exploration of lncRNA function will only continue to add to our appreciation of the complexity of transcriptional regulation, especially within the context of the seemingly endlessly complex development of the nervous system.

## Conflict of Interest Statement

The authors declare that the research was conducted in the absence of any commercial or financial relationships that could be construed as a potential conflict of interest.
